# Constructing Ni–Pt Bimetallic Catalysts for Catalytic Hydrogenation and Rearrangement of Furfural into Cyclopentanone with Insight in H/D Exchange by D_2_O Labeling

**DOI:** 10.1021/acsomega.4c02827

**Published:** 2024-06-21

**Authors:** Aurucha Kittisabhorn, Imtiaz Ahmed, Warangkana Pornputtapitak, Sakhon Ratchahat, Weerawut Chaiwat, Wanida Koo-amornpattana, Wantana Klysubun, Wanwisa Limphirat, Suttichai Assabumrungrat, Atthapon Srifa

**Affiliations:** † Department of Chemical Engineering, Faculty of Engineering, Mahidol University, Nakhon Pathom 73170, Thailand; ‡ Synchrotron Light Research Institute, Nakhon Ratchasima 30000, Thailand; § Center of Excellence in Catalysis and Catalytic Reaction Engineering, Department of Chemical Engineering, Faculty of Engineering, Chulalongkorn University, Bangkok 10330, Thailand; ∥ Bio-Circular-Green-Economy Technology & Engineering Center, Department of Chemical Engineering, Faculty of Engineering, Chulalongkorn University, Bangkok 10330, Thailand

## Abstract

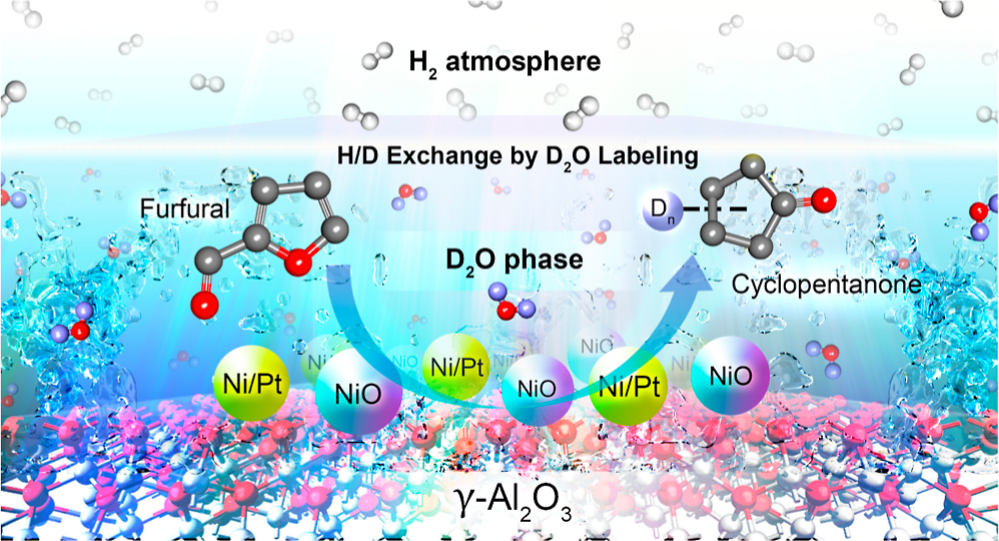

Developing a metallic catalyst for converting furfural (FAL) to highly valuable products such as cyclopentanone (CPO) is important for fine chemical synthesis by the efficient utilization of biomass resources. The presence of diverse unsaturated carbon atoms in FAL and the rearrangement of oxygen atoms hinder the production of CPO. We developed an optimal nickel (Ni)-to-platinum (Pt) molar ratio (1:0.007) for a bimetallic Ni–Pt/alumina (Al_2_O_3_) catalyst with a low Pt loading via an impregnation method to efficiently catalyze the selective hydrogenation of FAL in an aqueous solution to form CPO. The comprehensive characterizations by X-ray diffraction and X-ray absorption near edge structure analyses elucidated the formation of Ni^0^/Pt^0^ and Ni^2+^/Pt^4+^ after reduction by H_2_. The addition of a low amount of the Pt–Ni/Al_2_O_3_ catalyst resulted in an alleviation of H_2_ reduction behavior detected by hydrogen temperature-programmed reduction, accompanied by low H_2_ desorption ability observed by hydrogen temperature-programmed desorption. The catalytic activity of Ni–Pt/Al_2_O_3_ was higher than those of Ni/Al_2_O_3_ and Pt/Al_2_O_3_ catalysts. The maximum CPO yield was 66% with 93% FAL conversion under the optimized conditions (160 °C, 20 bar of H_2_ pressure, and 2 h). Isotopic deuterium oxide (D_2_O) labeling revealed the transfer of deuterium (D) atoms from D_2_O to the intermediates and products during hydrogenation and rearrangement, which confirmed that water was a medium for rearrangement and the source of hydrogen for the reaction. This study developed an efficient catalyst for the catalytic hydrogenation and ring rearrangement of FAL into CPO.

## 1 Introduction

**Figure 1:**
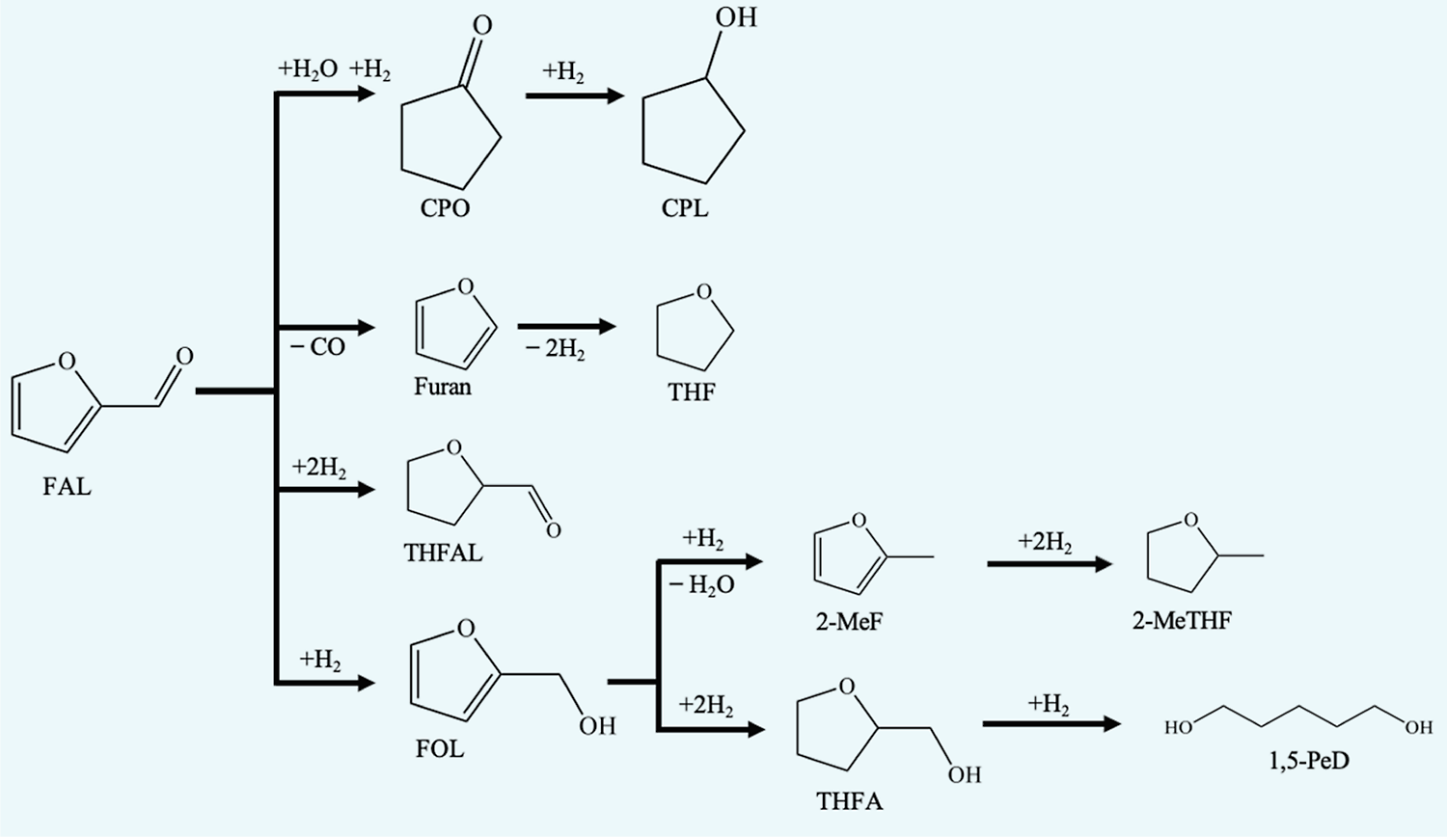
Reaction pathway of FAL hydrogenation into valuable products.

A sustainable and economic route to obtain high value-added chemicals via biomass biorefining has attracted extensive attention. The development of renewable biomass energy is crucial for resolving energy and ecology issues^1,2^ owing to the escalating depletion of fossil fuel sources.^3^ Biomass valorization has emerged as a potential process to meet the increasing demand for fuel and chemicals.^4,5^ Metal catalysts with scale-up strategies are crucial for the catalysis of biomass-derived molecules.^6^ Furfural (FAL), a biomass-derived resource rich in oxygen, is obtained via the dehydration of xylose. The hydrogenation of FAL produces furfuryl alcohol (FOL), tetrahydrofurfural (THFAL), tetrahydrofurfuryl alcohol (THFA), furan, tetrahydrofuran (THF), cyclopentanone (CPO), cyclopentanol (CPL), 2-methylfuran (2-MeF), 2-methyltetrahydrofuran (2-MeTHF), and pentanediols (PeDs) (Figure 1).^7−10^ The potential challenge is to attain the highest selectivity of the desired product via FAL hydrogenation with a multiproduct formation capability. The hydrogenative ring rearrangement of biomass-derived FAL to produce CPO has been extensively studied.^11^ CPO and CPL obtained through the selective hydrogenation and rearrangement of FAL are important precursors of various flavor-based compounds, pharmaceuticals, pesticides, electronic components, etc.^12,13^ CPO is a building block for fungicides, rubber-based chemicals, pharmaceuticals, and flavor- and fragrance-based compounds.^14^ It can also be used for the synthesis of polyamides, showcasing its potential for industrial applications. Alternately, the CPO intermediate is important for the synthesis of pharmaceuticals, insecticides, dyes, herbicides, and fragrances. The production of CPO from FAL was enhanced in the presence of water as a solvent,^15^ and the hydrogenative ring rearrangement was catalyzed by metals.^16^ Noble transition metal-based catalysts, especially platinum (Pt), were used for heterogeneous hydrogenation.^17^

Highly efficient nickel (Ni)-based catalysts were used for a sustainable and cost-effective approach to produce CPO via FAL hydrogenation. Ni–nickel oxide (NiO)/titanium oxide (TiO_2_) and Ni nanoparticles encapsulated in carbon (C) layers (Ni@NP-C) were used to catalyze the selective hydrogenation of FAL to CPO in water at temperature <150 °C.^18,19^ Hronec and Fulajtarová successfully synthesized CPO (76.50 mol % yield) by reacting FAL with hydrogen catalyzed by 5 wt % platinum (Pt)/C. Despite conducting aqueous hydrogenation at a relatively low temperature (160 °C), the industrial applicability of this method might be hindered by the scarcity of noble metals.^20^ Mironenko et al. investigated the mechanism of FAL hydrogenation in water catalyzed by palladium (Pd)/C. FAL hydrogenation proceeds through four pathways involving various acid-catalyzed furan ring opening and reduction reactions.^12^ However, the possible reaction mechanism of the hydrogenative rearrangement of FAL in water is still not clear.

Herein, we attempted to incorporate Pt and Ni on a γ-alumina (γ-Al_2_O_3_) support to synthesize the Pt–Ni/Al_2_O_3_ catalyst via an impregnation method using various Ni-to-Pt molar ratios. The prepared catalysts were characterized via nitrogen (N_2_) sorption, X-ray diffraction (XRD), X-ray absorption near edge structure (XANES), chemisorption, and transmission electron microscopy (TEM). The catalytic activity of Pt–Ni/Al_2_O_3_ for FAL hydrogenation to CPO in an aqueous phase was higher than those of the Ni/Al_2_O_3_ and Pt/Al_2_O_3_ catalysts. The isotopic deuterium oxide (D_2_O) labeling was investigated to comprehend the possible reaction mechanism.

## 2 Experimental Section

### 2.1 Synthesis of Catalysts

The bimetallic Ni–Pt-supported Al_2_O_3_ (Ni–Pt/Al_2_O_3_) catalysts with different molar ratios of Ni to Pt were prepared via a conventional wetness impregnation method (Scheme S1). Nickel(II) nitrate hexahydrate [Ni(NO_3_)_2_·6H_2_O; CARLO ERBA, purity ≥98.5%] and chloroplatinic acid solution (H_2_PtCl_6_; Sigma-Aldrich, 8 wt % in H_2_O) were used as the metal salts. Al_2_O_3_ used as a support was obtained from the Sasol Company, Germany. A mixture of an aqueous solution of Ni(NO_3_)_2_·6H_2_O and H_2_PtCl_6_ was added to an Al_2_O_3_ support. The obtained catalyst was dried at 110 °C for 6 h and calcined in stagnant air at 500 °C for 5 h at a heating rate of 5 °C min^–1^. The calcined catalyst was reduced at 500 °C for 3 h under a flow of pure hydrogen. The synthesized bimetallic Ni–Pt/Al_2_O_3_ catalysts with different Ni/Pt molar ratios of 1:0.004, 1:0.007, and 1:0.015 were denoted as Ni_1_Pt_0.004_/Al_2_O_3_, Ni_1_Pt_0.007_/Al_2_O_3_, and Ni_1_Pt_0.015_/Al_2_O_3_, respectively. The samples were analyzed via inductively coupled plasma-optical emission spectrometry (ICP-OES). Monometallic Ni/Al_2_O_3_ and Pt/Al_2_O_3_ catalysts were prepared following the same procedure using Ni(NO_3_)_2_·6H_2_O and H_2_PtCl_6_ precursors, respectively.

### 2.2 Characterization of Catalysts

The total pore volume, specific surface area, and average pore diameter of the synthesized catalysts were characterized via nitrogen desorption–adsorption at −196 °C using a gas sorption analyzer (Autosorb iQ Station 2). The undesired volatile substances were removed before analysis by pretreating the samples under a vacuum at 200 °C for 12 h. The phase and crystallinity were analyzed via XRD using an X-ray diffractometer (D8 ADVANCE, Bruker, Ltd.) with Cu Kα X-ray source radiation in the range of 2θ from 10 to 80° within a step time of 0.5 s at 40 kV and 30 mA. XANES analysis was used to study the change in the oxidation states of the reduced catalysts. The XANES spectra at the Ni K-edge and Pt L_3_-edge were obtained using transmission and fluorescent modes, respectively, using a Ge(220) monochromator crystal for scanning photon energy at Beamline 8 at the Synchrotron Light Research Institute, Thailand. The calibration of the photon energy was performed using Ni and Pt foil with reference K-edge and L_3_-edge energies of 8979 and 10,535 eV, respectively. The fluorescent mode of the Pt L_3_-edge was implemented by the investigation of the self-absorption effect using a platinum oxide (PtO_2_) standard. The linear combination fitting (LCF) and normalization of the XANES spectra were performed using the ATHENA program. The reducibility and metal–support interaction of catalysts were observed via hydrogen temperature-programmed reduction (H_2_-TPR) using a chemisorption analyzer (Quantachrome TPRWin v4.10). The calcined catalysts were pretreated at 200 °C for 1 h under an argon (Ar) flow rate of 50 cm^3^ min^–1^. The H_2_-TPR profiles were recorded using a thermal conductivity detector (TCD) under a flow of 10 vol % H_2_/Ar in a temperature range of 100–900 °C with a heating rate of 10 °C min^–1^. The acidity of the catalyst was observed via ammonia temperature-programmed desorption (NH_3_-TPD) using a similar system as that used for H_2_-TPR. The calcined catalysts were reduced in situ at 500 °C for 2 h at a rate of 10 °C min^–1^ under a 10 vol % H_2_/Ar flow rate of 50 cm^3^ min^–1^. Five vol % ammonia/helium (He) catalysts were adsorbed at 50 °C for 2 h accompanied by unabsorbed NH_3_ removal using an inert gas. The signal of NH_3_ desorption was recorded in the range of 50–1000 °C at a ramp temperature of 10 °C min^–1^ via TCD under a He gas. The ability of H_2_ adsorption–desorption was explored via hydrogen TPD (H_2_-TPD). The calcined catalyst was reduced in situ at 500 °C for 2 h at a heating temperature of 10 °C min^–1^ under a 10 vol % H_2_/Ar flow rate of 50 cm^3^ min^–1^. The catalysts were adsorbed with 10 vol % H_2_/Ar at 50 °C for 2 h, and the desorption of H_2_ was recorded in the temperature range of 50–1000 °C with a ramp temperature of 10 °C min^–1^ via TCD under He gas. The metal composition of the calcined catalysts was analyzed via ICP-OES (PerkinElmer, NexION 2000). The calcined catalyst was digested using a mixed acid solution of hydrochloric acid (HCl, 37% AR. grade; QReC, New Zealand) and nitric acid (HNO_3_, 65% AR. grade; QReC, New Zealand) at a volume ratio of 3:1 under microwave irradiation before the ICP-OES measurement. The metal species distribution and morphology of reduced Ni–Pt_7_/Al_2_O_3_, Ni/Al_2_O_3_, and Pt/Al_2_O_3_ were observed via high-resolution TEM (HR-TEM; JEOL/JEM-218 2100Plus). Finally, the weight loss of the reduced and used catalysts in the air was analyzed via thermogravimetric analysis (TGA; TGA 5500) in the temperature range of 30–1000 °C with a heating rate of 10 °C min^–1^.

### 2.3 Procedure for the Catalytic Conversion of Furfural

The hydrogenation and rearrangement of FAL were performed in a 100 mL batch reactor (Scheme S2). FAL (1 g, C_5_H_4_O_2_; Sigma-Aldrich, purity 99%), 40 mL of deionized water, and 0.1–0.3 g of reduced catalyst were loaded into a reactor. The reactor was flushed with H_2_ gas three times to remove the inside air and was subsequently pressurized to the desired operating pressure. The reaction temperature was maintained at the desired condition using a temperature controller under stirring at 600 rpm. The reactor was placed in cool water to quench the reaction. The products were collected and separated via filtration. The obtained liquid product was quantitatively analyzed via gas chromatography (GC) equipped with a flame ionization detector (FID) (GC 2014, Shimadzu) and a CP-Wax 52 CB column (30 m × 0.25 mm × 0.25 μm). The operating conditions for GC-FID are summarized in Table S1. The FAL conversion and product yields (FOL, CPO, and CPL) were calculated using eqs 1 and 2.
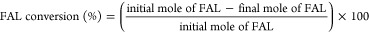
1
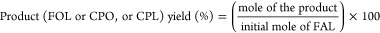
2

### 2.4 Isotopic D_2_O Labeling

The reaction mechanism of the hydrogenation and ring rearrangement of FAL to CPO was investigated via the isotopic D_2_O-labeling experiment and was compared with that conducted in water (H_2_O) as the solvent at 140 °C, 20 bar H_2_, and 2 h reaction time in the presence of reduced Ni_1_Pt_0.007_/Al_2_O_3_. The liquid product was extracted from the aqueous phase by using toluene and analyzed via GC–mass spectrometry (MS) (7890A, Agilent Technologies).

## 3 Results and Discussion

### 3.1 Catalyst Characterization

#### 3.1.1 N_2_ Sorption and ICP-OES Analysis

Type IV isotherms with H1-type hysteresis loops are observed in the adsorption–desorption isotherm of the calcined catalysts, confirming the appearance of mesoporous material (Figure S1; Table 1).^21^ The Brunauer–Emmett–Teller (BET) surface area of the pristine Al_2_O_3_ support is 194 m^2^ g^–1^ with a pore volume (*V*_p_) of 0.50 cm^3^ g^–1^.^22^ The BET surface area of calcined Ni/Al_2_O_3_ is 187 m^2^ g^–1^ with a total *V*_p_ of 0.50 m^2^ g^–1^, whereas the BET surface area of calcined Pt/Al_2_O_3_ is 207 m^2^ g^–1^ with a total *V*_p_ of 0.51 m^2^ g^–1^. Thus, the porosity of Al_2_O_3_ is modified by the addition of Pt.^23^ The BET surface area of Pt–Ni/Al_2_O_3_ is reduced by ∼12.7 to 20.6% than that of Ni/Al_2_O_3_ either owing to the blockage or converging of pores during metal loading. The *V*_p_ of the highest Pt-loaded Ni_1_Pt_0.015_/Al_2_O_3_ is reduced. However, the average pore size diameters of Ni_1_Pt_0.004_/Al_2_O_3_, Ni_1_Pt_0.007_/Al_2_O_3_, and Ni_1_Pt_0.015_/Al_2_O_3_ are nearly similar, indicating the partial coverage of metal components on the external area of the Al_2_O_3_ support. The average pore size analyzed via the Barret–Joyner–Halenda (BJH) method is 7.4 nm for all of the calcined catalysts. The loaded Ni content is 10.2 ± 0.9 wt % for all the Ni-containing catalysts, while the loaded Pt content is low (0.126–0.584 wt %) for Ni_1_Pt_0.004_/Al_2_O_3_, Ni_1_Pt_0.007_/Al_2_O_3_, and Ni_1_Pt_0.015_/Al_2_O_3_, as analyzed via ICP-OES (Table 1).

**Table 1:** Porosity Obtained via N_2_ Sorption Experiments and Metal Composition Obtained via ICP-OES of the Calcined Catalysts^a^

catalyst	N_2_ sorption		metal content^d^			Pt/Ni^d^ mole ratio
	*S*_BET_ (m^2^ g^–1^)^a^	*V*_p_ (cm^3^ g^–1^)^b^	*D*_p_ (nm)^c^	Ni (wt %)	Pt (wt %)	
Ni/Al_2_O_3_	187	0.50	7.4	9.69	0	
Pt/Al_2_O_3_	207	0.51	7.4	0	10.58	
Ni_1_Pt_0.004_/Al_2_O_3_	164	0.42	7.4	9.6	0.126	0.004
Ni_1_Pt_0.007_/Al_2_O_3_	148	0.40	7.4	10.16	0.242	0.007
Ni_1_Pt_0.015_/Al_2_O_3_	149	0.38	7.4	11.54	0.584	0.015

a *S*_BET_ obtained from the adsorption branch of the N_2_ isotherm.

b *V*_p_ calculated from N_2_ adsorption at a relative pressure of ∼0.990.

c *D*_p_ obtained from the desorption branch via the BJH method.

d Metal composition determined via ICP-OES analysis.

#### 3.1.2 XRD Analysis

**Figure 2:**
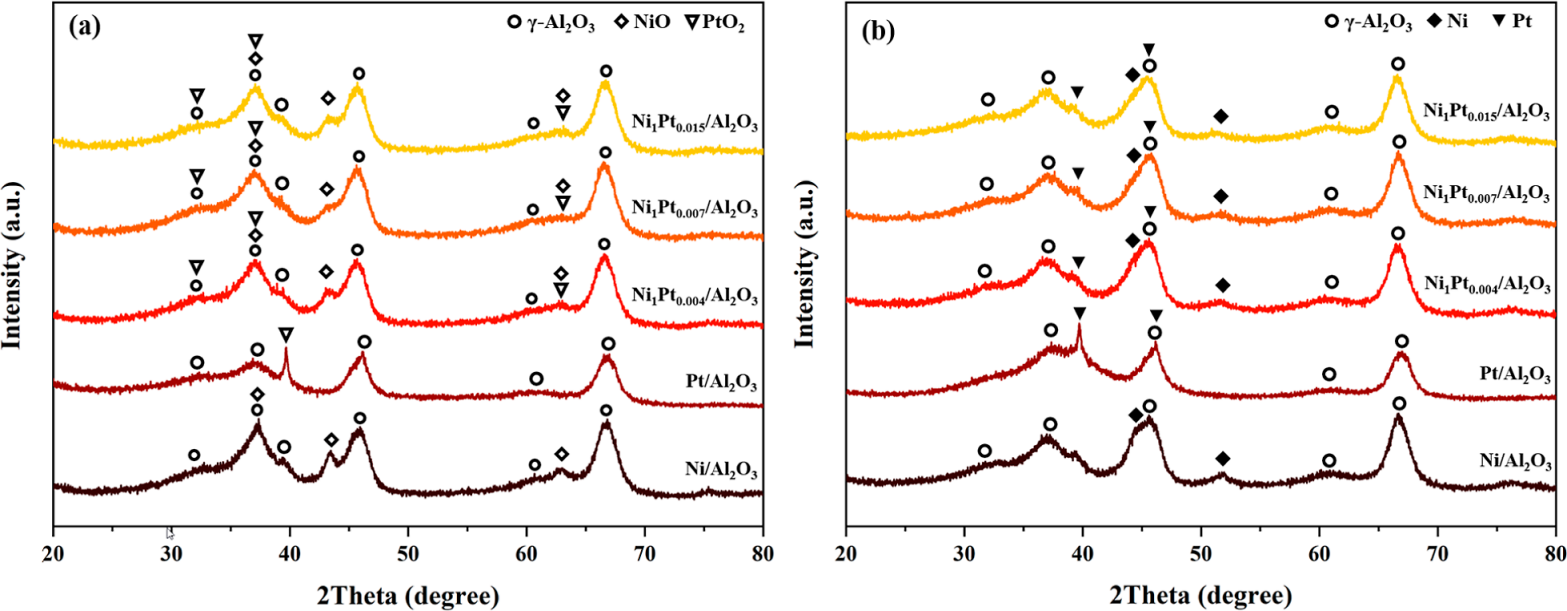
XRD patterns of (a) calcined and (b) reduced Ni/Al_2_O_3_, Pt/Al_2_O_3_, Ni_1_Pt_0.004_/Al_2_O_3_, Ni_1_Pt_0.007_/Al_2_O_3_, and Ni_1_Pt_0.015_/Al_2_O_3_.

The phase purity and crystallinity of all of the reduced and calcined Ni/Al_2_O_3_, Pt/Al_2_O_3_, Ni_1_Pt_0.004_/Al_2_O_3_, Ni_1_Pt_0.007_/Al_2_O_3_, and Ni_1_Pt_0.015_/Al_2_O_3_ were analyzed via XRD (Figure 2). The XRD patterns of the catalysts show the characteristic 2θ peaks at 19, 32, 38, 39, 45, and 67° corresponding to (101), (112), (103), (202), (220), and (224) planes, respectively, which are attributed to the Al_2_O_3_ phase (PDF 01-074-4629).^22,24−27^Figure 2a indicates the NiO phase (PDF 01-080-5508) of calcined Ni/Al_2_O_3_ and Ni–Pt/Al_2_O_3_ with the diffraction peaks at 37, 44, and 63°, indicating the (111), (200), and (220) planes, respectively.^22^ The peaks of calcined Pt/Al_2_O_3_ and Ni–Pt/Al_2_O_3_ at 32, 37, and 63° correspond to the (111), (222), and (311) planes, respectively, ascribed to the PtO_2_ phase (PDF 01-089-3603). The peaks of reduced Ni/Al_2_O_3_ and Ni–Pt/Al_2_O_3_ (Figure 2b) at 44 and 52° correspond to metallic Ni(111) and (200) planes, respectively (PDF 00-004-0850).^22^ Alternately, the peaks at 39 and 46° of reduced Pt/Al_2_O_3_ and Ni–Pt/Al_2_O_3_ corresponding to (111) and (200) planes, respectively, are ascribed to the metallic Pt phase (PDF 00-004-0802).^25−27^ Thus, NiO and PtO_2_ were reduced to Ni and Pt under H_2_ at 500 °C before the catalytic performance.

#### 3.1.3 XANES Analysis

**Figure 3:**
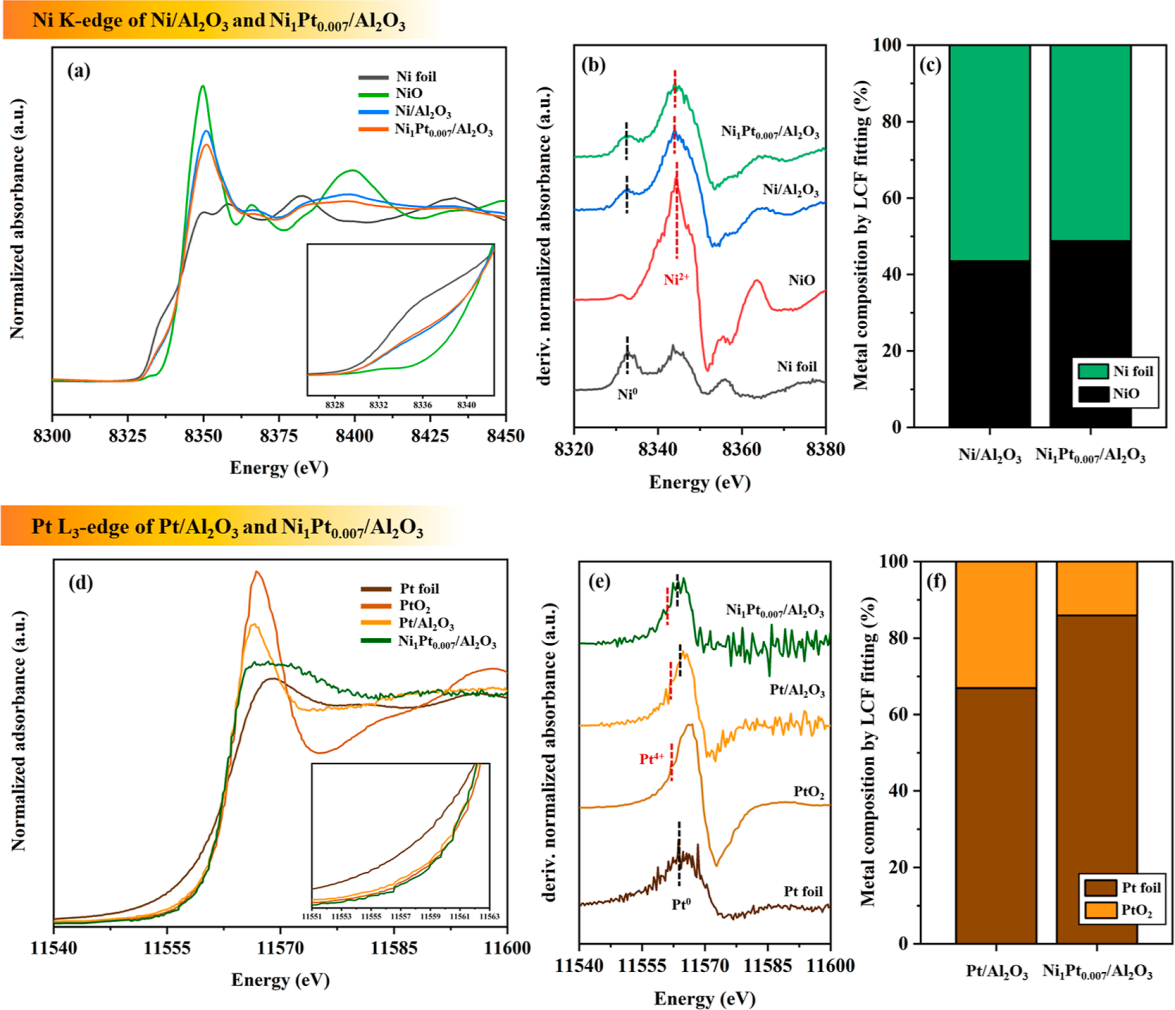
(a,d) Normalized XANES spectra, (b,e) first derivative, and (c,f) metal composition via LCF fitting of (a–c) Ni K-edges and (d–f) Pt L_3_-edges of reduced Ni/Al_2_O_3_ and Ni_1_Pt_0.007_/Al_2_O_3_ containing all combinations of Ni and Pt standards.

The reduced Ni_1_Pt_0.007_/Al_2_O_3_ was selected and analyzed via XANES because of its highest activity for CPO production among the three synthesized bimetallic catalysts. Figure 3 illustrates the results of XANES for the Pt L_3_-edge and Ni K-edge of reduced Ni_1_Pt_0.007_/Al_2_O_3_ and Ni/Al_2_O_3_ with the probable Ni and Pt standards. The valence state of the reduced sample is transformed, as indicated by the edge energy, oscillation shape, white line peak, and first derivative spectra. The Ni K-edge region (Figure 3a) shows that Ni/Al_2_O_3_ and Ni_1_Pt_0.007_/Al_2_O_3_ peaks shift to low energy with a decrease in white line intensity than that of the NiO standard, indicating a partial reduction of NiO to Ni. The normalized first derivative of the XANES spectra confirms that Ni of reduced Ni/Al_2_O_3_ and Ni_1_Pt_0.007_/Al_2_O_3_ exists as Ni^2+^ and metallic Ni^0^ owing to the similar shape and edge position of Ni foil and NiO standards (Figure 3b). LCF (Figure 3c) shows the coexistence of NiO and metallic Ni^0^ in the reduced Ni/Al_2_O_3_ and Ni_1_Pt_0.007_/Al_2_O_3_. The Ni^0^ fraction of Ni_1_Pt_0.007_/Al_2_O_3_ is higher than that of Ni/Al_2_O_3_ because of the H_2_ spillover effect from Pt to Ni. The Pt L_3_-edge results of reduced Pt/Al_2_O_3_ and Ni_1_Pt_0.007_/Al_2_O_3_ (Figure 3d) are similar to the Pt standard than that of the PtO_2_ standard. Thus, Pt^0^ is the major fraction of the reduced catalysts. The first derivative spectra (Figure 3e) of reduced Ni_1_Pt_0.007_/Al_2_O_3_ and Ni/Al_2_O_3_ show that the shift of the edge position is identical to that of the Ni foil standard. A higher Pt^0^ content (∼80%) of Ni_1_Pt_0.007_/Al_2_O_3_ is indicated via LCF compared to the lower Pt^0^ content (∼67%) of Pt/Al_2_O_3_ (Figure 3f). Thus, PtO_*x*_ strongly interacts with the Al_2_O_3_ support in the Pt/Al_2_O_3_ catalyst.

#### 3.1.4 Chemisorption Analysis

**Figure 4:**
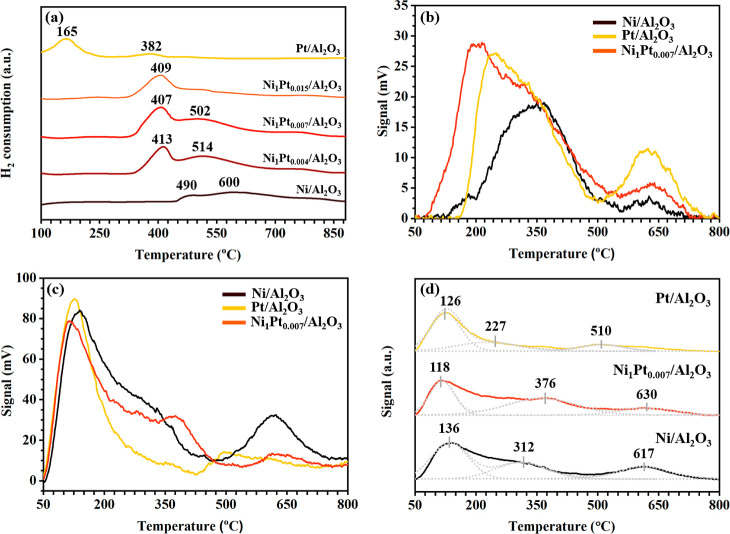
(a) H_2_-TPR of Ni/Al_2_O_3_, Pt/Al_2_O_3_, Ni_1_Pt_0.004_/Al_2_O_3_, Ni_1_Pt_0.007_/Al_2_O_3_, and Ni_1_Pt_0.015_/Al_2_O_3_. (b) H_2_-TPD and (c) NH_3_-TPD profiles with (d) deconvoluted NH_3_-TPD profiles of Ni/Al_2_O_3_, Pt/Al_2_O_3_, and Ni_1_Pt_0.007_/Al_2_O_3_.

The H_2_-TPR of the calcined catalysts was initially measured to investigate the catalyst reducibility and metal–support interactions (Figure 4a). The signal of H_2_ consumption of calcined Ni/Al_2_O_3_ was detected in the temperature range of 400−800 °C owing to the weak interaction of bulk NiO and the strong interaction of surface NiO with Al_2_O_3_.^28,29^ The H_2_-TPR profile of Pt/Al_2_O_3_ comprises two reduction peaks in the ranges of 100–300 and 350–450 °C, indicating the reduction of PtO_*x*_ and the strong interaction of PtO_*x*_ with Al_2_O_3_, respectively. The H_2_-TPR profiles of Ni–Pt/Al_2_O_3_ shift to a lower reduction temperature than that of Ni/Al_2_O_3_. Thus, the H_2_ spillover effect from Pt to Ni is pronounced after the addition of a low amount of Pt to Ni/Al_2_O_3_. Two major reduction peaks of Ni–Pt/Al_2_O_3_ in the ranges of 350–490 and 490–700 °C indicate the reduction of NiO and stable NiO interaction with Al_2_O_3_, respectively.

The effect of Pt addition was investigated on the ability of H_2_ adsorption–desorption, and the H_2_-TPD experiments were performed for reduced Ni/Al_2_O_3_, Pt/Al_2_O_3_, and Ni_1_Pt_0.007_/Al_2_O_3_ (Figure 4b). Two H_2_ desorption peaks of reduced Ni/Al_2_O_3_ are present in the ranges of 155–500 and 550–750 °C, whereas Pt/Al_2_O_3_ showed two peaks in the ranges of 200–500 and 500–775 °C. Thus, the H_2_ desorption ability of Pt/Al_2_O_3_ is weaker than that of Ni/Al_2_O_3_. The H_2_-TPD profile of Ni–Pt/Al_2_O_3_ comprises two wide peaks (80–500 and 540–800 °C) similar to that of Ni/Al_2_O_3_. Thus, the H_2_ desorption ability of Ni-containing catalysts is associated with weak and strong chemisorbed H_2_ in the dissociate states. H_2_ desorbed at a low temperature is the H_2_ adsorbed by metal particles, while H_2_ desorbed at a high temperature is from the strongly adsorbed H_2_ at the metal–support interface, which conforms with the reported behavior of Ni-based catalysts.^30,31^ The H_2_ desorption peak of Ni–Pt/Al_2_O_3_ shifts to a lower temperature than those of Ni/Al_2_O_3_ and Pt/Al_2_O_3_ with a low molar ratio of Pt to Ni, indicating that the interaction of H_2_ with Ni–Pt/Al_2_O_3_ is weakened by the addition of Pt to Ni catalysts.

The acidity of the catalysts was analyzed via NH_3_-TPD (Figure 4c,d). The NH_3_ desorption profiles displayed differences in the desorption temperature and peak intensity, indicating that the acidity affects the strength and quantity of the catalysts. The deconvoluted peaks were indicated as weak (50–240 °C), medium (320–450 °C), and strong (550–700 °C) acid sites (Figure 4d) in accordance with literature to distinguish the strengths of acids.^28^ The intensity of the Ni/Al_2_O_3_ peak for weak and strong acid sites is the highest among the other catalysts, whereas that of Pt/Al_2_O_3_ shows the lowest intensity. Thus, Ni incorporated into Al_2_O_3_ exhibits more acidic sites than Pt incorporated into Al_2_O_3_ (Figure 4c). The acidity and strength of Ni–Pt/Al_2_O_3_ decrease following the addition of Pt to Ni/Al_2_O_3_ owing to the decreasing number and strength of acid sites with a relative acidity distribution (Table S2). The moderate catalyst acidity based on the strength and quantity is positive for the hydrogenation and rearrangement of FAL to CPO.

#### 3.1.5 TEM Results

**Figure 5:**
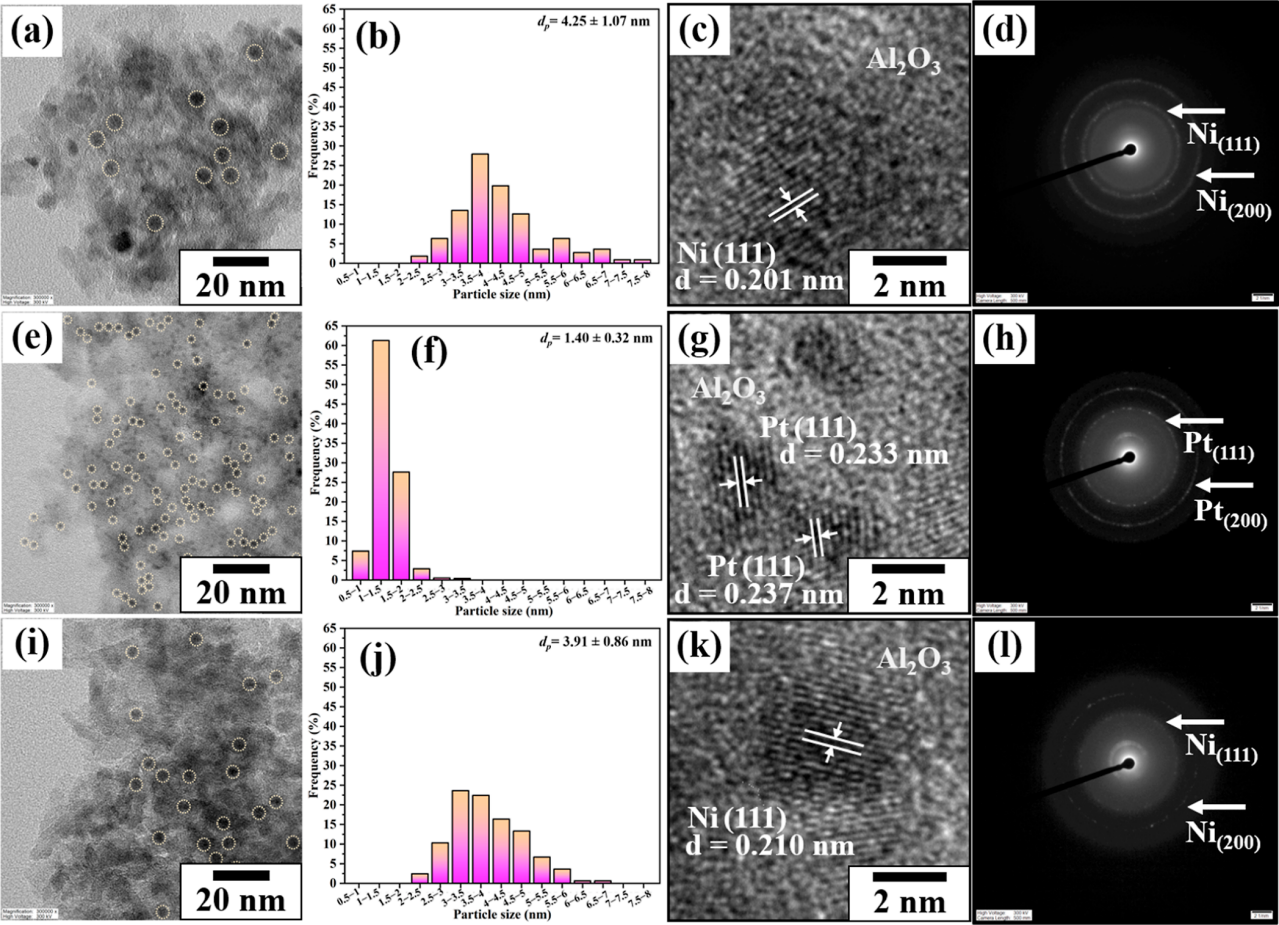
(a,e,i) TEM images, (b,f,j) particle size distribution, and (c,g,k) HR-TEM images with (d,h,l) diffraction patterns of reduced (a–d) Ni/Al_2_O_3_, (e–h) Pt/Al_2_O_3_, and (i–l) Ni_1_Pt_0.007_/Al_2_O_3_.

The TEM results of reduced Ni_1_Pt_0.007_/Al_2_O_3_ were compared with those of Ni/Al_2_O_3_ and Pt/Al_2_O_3_ to observe the particle size and distribution morphology (Figure 5). Metal particles were detected as dark spots on Al_2_O_3_ with different metal particle sizes. The average particle size of Ni/Al_2_O_3_ is 4.25 ± 1.07 nm (Figure 5a,b), while Pt/Al_2_O_3_ exhibits a small metal particle size of 1.40 ± 0.32 nm (Figure 5e,f). The particle size of Ni_1_Pt_0.007_/Al_2_O_3_ (3.91 ± 0.86 nm) is lower than that of Ni/Al_2_O_3_ (Figure 5i,j). Additionally, d-spacing (Figure 5c,k) with the diffraction patterns (Figure 5d,l) of Ni-containing catalysts was obtained via HR-TEM, which indicates the (111) lattice plane of metallic Ni particles with a spacing of 0.201–0.210 nm.^32,33^ Thus, the formation of Ni^0^ after reduction by H_2_ is confirmed. The d-spacing (Figure 5g) of Pt/Al_2_O_3_ with the diffraction pattern (Figure 5h) indicates the (111) lattice plane of metallic Pt with a spacing of 0.233–0.237 nm,^34−36^ indicating the formation of Pt^0^ after H_2_ reduction.

### 3.2 Evaluation of Catalytic Performance

#### 3.2.1 Effect of the Ni-to-Pt Molar Ratio

**Figure 6:**
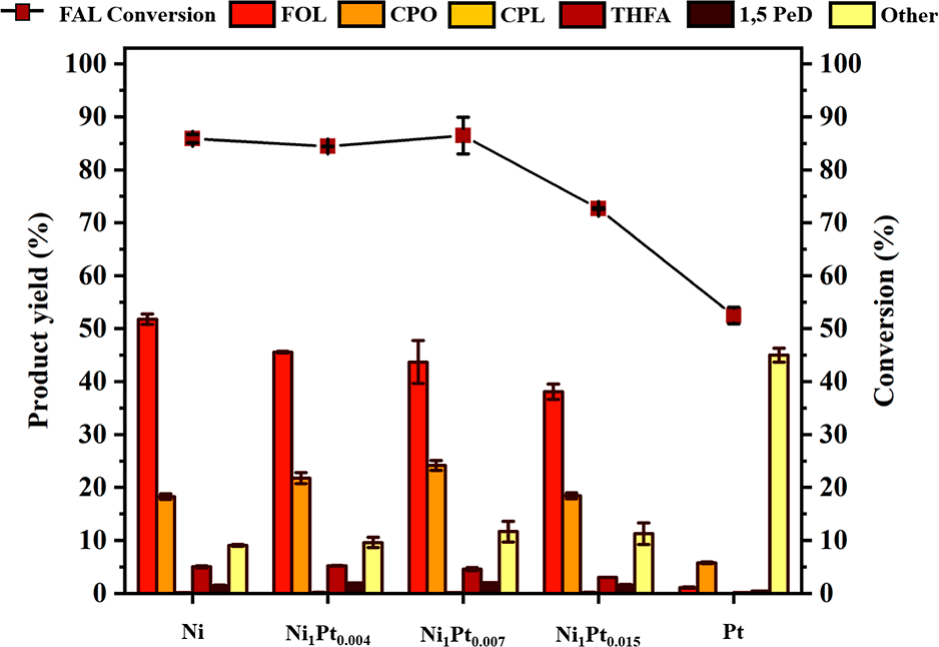
FAL conversion and product yield catalyzed by different catalysts at 140 °C, 20 bar H_2_, and 2 h reaction time. The catalyst loading was 20 wt % based on the initial mass of FAL. The experiments were conducted using 1 g of FAL feedstock in 40 g of water with 20% loading of the catalyst based on the initial mass of FAL.

The catalytic activity of Ni–Pt/Al_2_O_3_ with different molar ratios (1:0.004–1:0.015) of Ni to Pt was investigated and compared with that of Ni/Al_2_O_3_ and Pt/Al_2_O_3_ at 140 °C, 20 bar H_2_, and 2 h reaction time (Figure 6). FAL conversion to CPO is <100% in the presence of the examined catalysts, and Ni–Pt/Al_2_O_3_ demonstrates superior catalytic performance. The conversion (85%) of FAL catalyzed by Ni/Al_2_O_3_ is similar to that by Ni_1_Pt_0.004_/Al_2_O_3_ and Ni_1_Pt_0.007_/Al_2_O_3_. An increase in the Ni-to-Pt molar ratio of up to 1:0.015 (Ni_1_Pt_0.015_/Al_2_O_3_) decreases FAL conversion. The lowest FAL conversion (52.5%) was obtained in the presence of Pt/Al_2_O_3_. The highest yield of CPO in the presence of Ni_1_Pt_0.007_/Al_2_O_3_ is 24.2%, while an 18.3% yield was obtained in the presence of Ni/Al_2_O_3_. Thus, a low amount of Pt improves the hydrogenation and rearrangement of FAL to CPO at a low FAL conversion level. Pt/Al_2_O_3_ catalyzed the FAL conversion to form CPO in a 5.8% yield with the generation of undesired or intermediate products up to 45%. Thus, the optimal Ni_1_Pt_0.007_/Al_2_O_3_ catalyst was selected for further investigation.

#### 3.2.2 Effect of Operating Conditions

**Figure 7:**
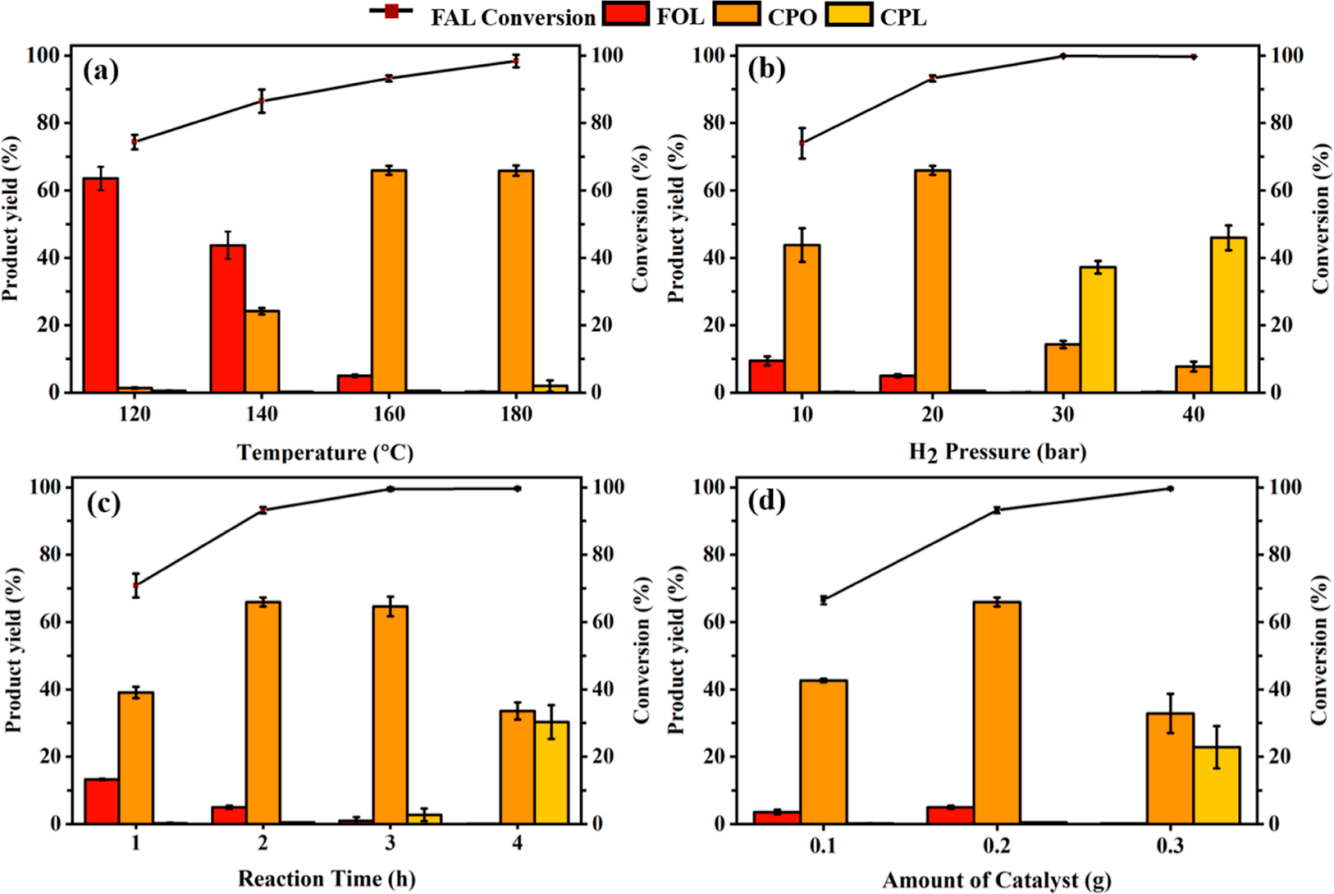
Effects of the reaction temperature, reaction time, H_2_ pressure, and catalyst loading of FAL conversion catalyzed by Ni_1_Pt_0.007_/Al_2_O_3_. Reaction conditions are (a) 20% catalyst, 20 bar of H_2_ pressure, and 2 h reaction time; (b) 20% catalyst, 160 °C, and 2 h; (c) 20% catalyst, 160 °C, and 20 bar; and (d) 160 °C, 20 bar, and 2 h. The experimental measurements were conducted using 1 g of FAL feedstock in 40 g of water.

The effects of the reaction temperature, reaction time, H_2_ pressure, and catalyst loading were systematically investigated to obtain the highest yield of CPO catalyzed by Ni_1_Pt_0.007_/Al_2_O_3_ (Figure 7). The effect of the reaction temperature in the range of 120–180 °C under constant parameters (20 bar H_2_ pressure and 2 h reaction time) was explored. Figure 7a shows that FAL conversion increases with the reaction temperature. The CPO yield increased from 120 to 160 °C and remained stable (∼66% yield) at 160 and 180 °C. FOL was formed as an intermediate at low temperatures, which disappeared at high temperatures. Thus, the rearrangement of FAL and hydrogenation to form FOL followed by the formation of CPO are catalyzed at high temperatures. The highest (66%) CPO yield formed via 93% of FAL conversion was obtained at 160 °C. The effect of H_2_ pressure of 10–40 bar was examined to facilitate the solubility of H_2_ in an aqueous system at the optimum temperature (160 °C). Figure 7b shows an increase in FAL conversion in the H_2_ pressure from 10 to 30 bar, which becomes 100% at 40 bar. The maximum CPO yield was achieved at 20 bar. A further increase in the H_2_ pressure to 30 bar formed CPL via the direct hydrogenation of CPO. The effect of the reaction time was evaluated from 1 to 4 h under optimized 20 bar H_2_ pressure at 160 °C (Figure 7c). The highest FAL conversion and CPO yield were obtained by increasing the reaction time. At the initial stage (1–2 h), FA was preliminarily generated via direct FAL hydrogenation. An extended reaction time of 2–3 h induced the rearrangement of FOL to produce CPO, which further produced CPL by the hydrogenation of the C=O group of CPO by increasing the reaction time to 4 h. The catalyst amount was varied from 0.1 to 0.3 g to obtain 10, 20, and 30% catalyst loading based on the initial mass of FAL (Figure 7d). The highest FAL conversion and CPO yield were obtained with a catalyst loading of 20% of FAL feedstock. Increasing catalyst loading to 30% formed CPL via CPO hydrogenation. The maximum yield of CPO was 66% with 93% FAL conversion in the optimal conditions of 20% catalyst, 160 °C, 20 bar H_2_, and 2 h reaction time.

#### 3.2.3 Catalyst Reusability

The reusability experiment of the optimal Ni_1_Pt_0.007_/Al_2_O_3_ was evaluated for three consecutive cycles under the optimal conditions (140 °C, 20 bar H_2_ pressure, and 2 h reaction time) (Figure S2). The catalyst used was separated from the reaction mixture and reactivated via calcination and reduction. The CPO yield and FAL conversion decreased after three consecutive experiments, indicating that the catalyst was deactivated during the reaction (Figure S2a). The XRD results of the used catalyst show no remarkable alteration of the catalyst structure before and after the reaction (Figure S2b). The used Ni_1_Pt_0.007_/Al_2_O_3_ was analyzed via TGA under a supply of air to estimate the amount of deposited carbon (Figure S2c). The catalyst lost weight at <200 °C owing to the removal of moisture. The combustion of carbon or an intermediate deposit was detected at >280 °C. The used Ni_1_Pt_0.007_/Al_2_O_3_ exhibited only 20% weight loss. Therefore, carbon formation is the primary factor for catalyst deactivation during the catalytic hydrogenation and rearrangement of FAL to CPO.

### 3.3 Isotopic D_2_O Labeling

**Figure 8:**
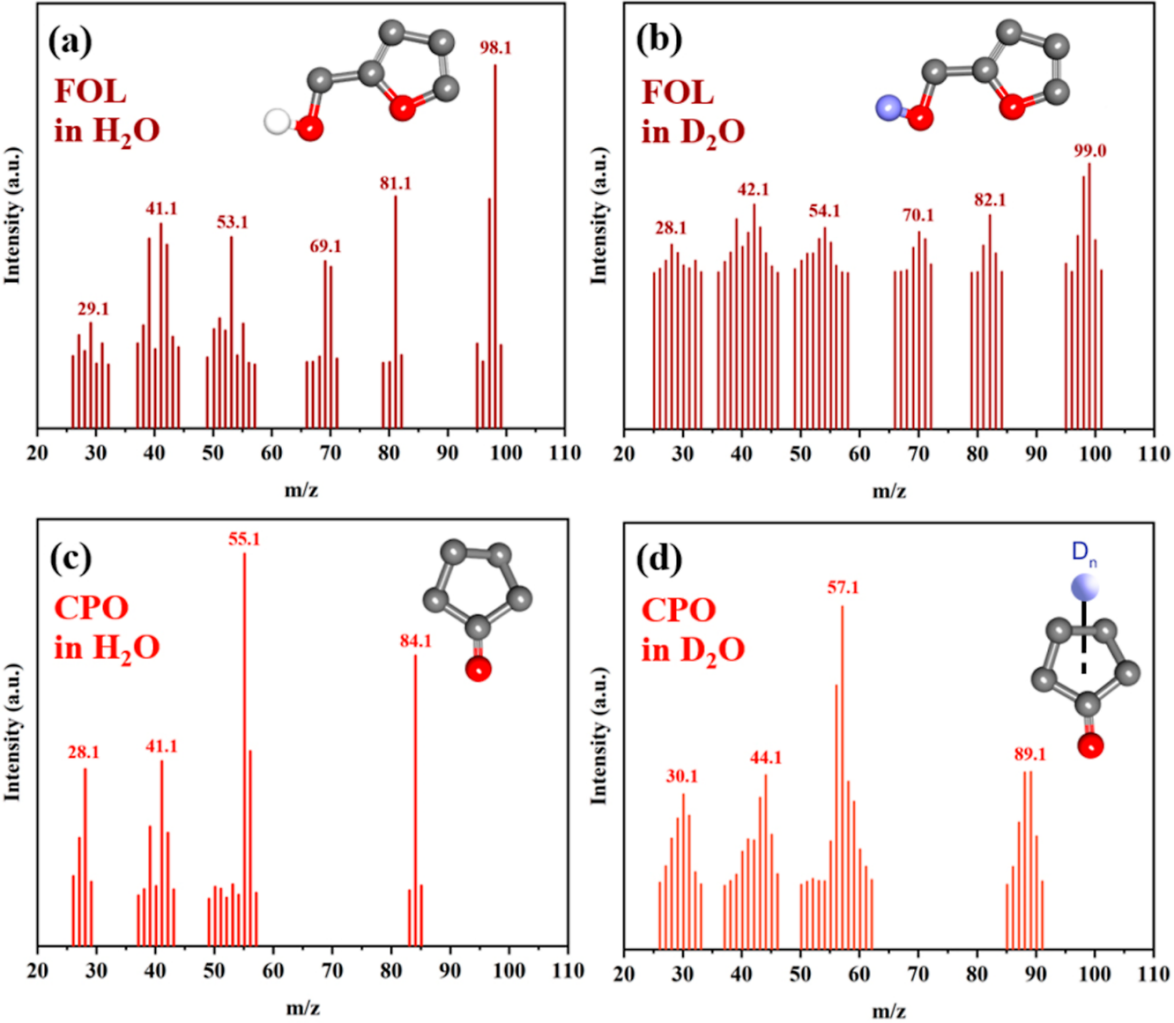
Mass spectra of FOL and CPO fragmentation patterns during the catalytic hydrogenation and rearrangement of FAL in (a,c) H_2_O and (b,d) D_2_O at 140 °C and 20 bar H_2_ pressure for 2 h. The catalyst loading was 20 wt % based on the initial mass of FAL.

The reaction mechanism of the hydrogenation and ring rearrangement of FAL to CPO was investigated via isotopic D_2_O labeling and was compared with another set of reactions performed in water as the solvent under the optimal conditions (140 °C, 20 bar H_2_ pressure, and 2 h reaction time). Figure 8a,c shows the GC–MS peaks of FOL (*m*/*z* = 98) and CPO (*m*/*z* = 84). FOL (*m*/*z* = 99) and CPO (*m*/*z* = 89) were generated in D_2_O under H_2_ pressure (Figure 8b,d), indicating that deuterium (D) was substituted via an isotopic exchange between D from D_2_O and the H atom in FAL. Additionally, the reaction in an aqueous solution was performed without pressurized H_2_ gas under the optimized reaction condition to form 8.8% FAL conversion and 0% CPO yield, indicating that the reaction proceeds through the H_2_–D_2_O exchange process.

**Scheme 1:**
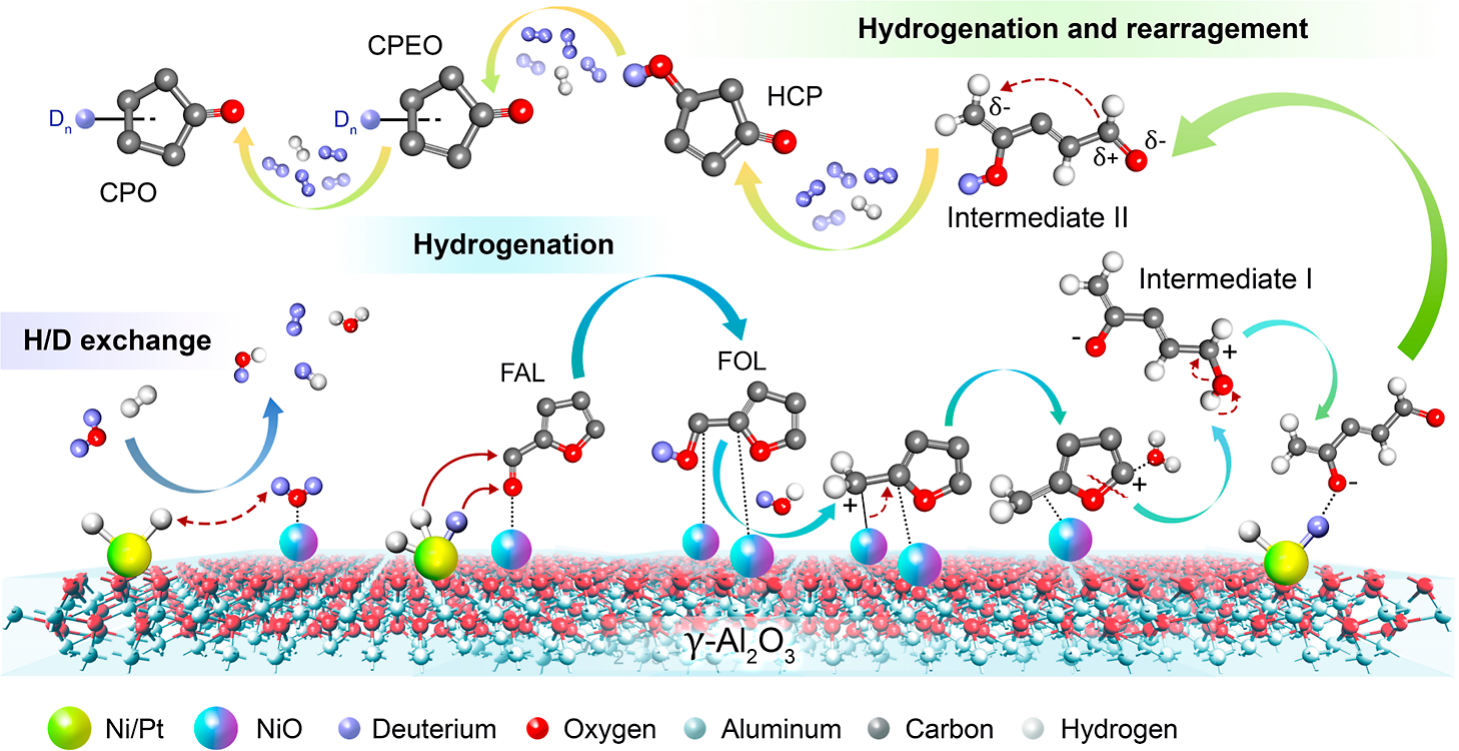
Schematic Representation of the Proposed Reaction Mechanism for the Catalytic Hydrogenation and Rearrangement of FAL into CPO Catalyzed by Ni–Pt/Al_2_O_3_ under Atmospheric D_2_O Solvent

Therefore, the proposed reaction mechanism is as described in Scheme 1.^37−40^ H_2_ gas is more preferentially adsorbed and dissociated by metallic active sites than oxophilic active sites.^23,28,41^ The XRD and XANES results indicate the coexistence of Ni^0^/NiO states and metallic Pt^0^ as prominent valence states after the reduction of Ni–Pt/Al_2_O_3_ by hydrogen. The adsorption and dissociation of H_2_ molecules occur at the metallic sites of Ni^0^/Pt^0^, whereas the oxophilic NiO sites are crucial for the C=O and C–OH adsorption of FAL and FOL, respectively. The H_2_–D_2_O exchange generates the minor products of HD, D_2_, HOD, and H_2_O.^37^ H_2_ or D_2_ is dissociated on the metallic sites of Ni^0^/Pt^0^ during hydrogenation to generate H or D atoms. The C=O group of FAL is preferentially adsorbed by the NiO sites. D or H atoms adsorbed by the metallic sites are transferred to the carbonyl group of FAL to produce FOL via direct hydrogenation. FOL is adsorbed by oxophilic NiO sites and forms a FOL carbocation with water. The carbocation rearranges via nucleophilic attack by water to generate Intermediate I. The oxygen anion of Intermediate I is attacked by H/D atoms to form Intermediate II. The ring closure of Intermediate II via 4π-conrotatory cyclization forms 4-hydroxy-2-cyclopentenone (HCP) after hydrogenation. 2-Cyclopentenone (CPEO) is generated via dehydration. Finally, the hydrogenation of CPEO generates CPO under a H_2_ or D_2_ atmosphere.^42−44^ The generated CPO in D_2_O shows an increased mass/charge ratio (*m*/*z*) than that in water, confirming the transfer of D atoms from D_2_O to intermediates and products during hydrogenation and rearrangement processes. This investigation confirms that water is a reactive solvent for the rearrangement process and a hydrogen source for the reaction.

## 4 Conclusions

A low Pt-loaded Ni–Pt/Al_2_O_3_ catalyst with different Ni-to-Pt molar ratios was successfully prepared via an impregnation method. Ni–Pt/Al_2_O_3_ catalyzed the hydrogenation of FAL to CPO in water, and the catalytic activity was compared with that of Ni/Al_2_O_3_ and Pt/Al_2_O_3_. The coexistence of Ni^0^/Pt^0^ and Ni^2+^/Pt^4+^ generated after reduction by H_2_ was elucidated via XRD and XANES. The H_2_ spillover effect from Pt to Ni was promoted after the addition of a low amount of Pt to Ni/Al_2_O_3_, as confirmed via H_2_-TPR. The addition of a low amount of Pt to Ni reduced the H_2_ desorption ability of Ni–Pt/Al_2_O_3_ than those of Ni/Al_2_O_3_ and Pt/Al_2_O_3_. The catalyst acidity decreased after the incorporation of Pt to Ni/Al_2_O_3_. Additionally, the particle size of Ni–Pt/Al_2_O_3_ was smaller than that of Ni/Al_2_O_3_. The maximum yield of CPO was 66% with 93% FAL conversion under the optimal conditions (160 °C, 20 bar H_2_, and 2 h) catalyzed by 20% of Ni_1_Pt_0.007_/Al_2_O_3_. Isotopic D_2_O labeling showed the transfer of D atoms in D_2_O to the intermediates and products during the hydrogenation and rearrangement processes, confirming that water was the solvent for rearrangement and H was the source for the reaction.

## Data Availability

All data related to the findings of this study are accessible from the corresponding author Atthapon Srifa on reasonable request.

## Supporting Information Available

Schematic illustration of catalyst preparation and the experimental setup; operating conditions of GC equipped with a FID; relative acidity distribution of reduced catalysts; N_2_ adsorption and desorption isotherms and pore size distribution; reusability experiment with XRD patterns; and TGA of reduced and spent catalysts (PDF)
