# Supplementary material for: Constructing Ni–Pt Bimetallic Catalysts for Catalytic Hydrogenation and Rearrangement of Furfural into Cyclopentanone with Insight in H/D Exchange by D2O Labeling
Source: ACS Omega. 2024 Jun 21;9(26):28637–47. doi: 10.1021/acsomega.4c02827 (PMC11223191; doi:10.1021/acsomega.4c02827)
Supplement: Supplementary file 1 — ao4c02827_si_001.pdf [file ao4c02827_si_001.pdf]

Supplementary Information

Constructing Ni–Pt Bimetallic Catalysts for Catalytic Hydrogenation and Rearrangement of Furfural into Cyclopentanone with Insight in H/D Exchange by D_2O Labeling

Aurucha Kittisabhorn[a], Imtiaz Ahmed[a], Warangkana Pornputtapitak[a], Sakhon Ratchahat[a], Weerawut Chaiwat[a], Wanida Koo-amornpattana[a], Wantana Klysubun[b], Wanwisa Limphirat[b], Suttichai Assabumrungrat[c,d], and Atthapon Srifa[a,*]

[a] Department of Chemical Engineering, Faculty of Engineering, Mahidol University, Nakhon Pathom 73170, Thailand

[b] Synchrotron Light Research Institute, Nakhon Ratchasima, 30000, Thailand

[c] Center of Excellence in Catalysis and Catalytic Reaction Engineering, Department of Chemical Engineering, Faculty of Engineering, Chulalongkorn University, Bangkok 10330, Thailand

[d] Bio-Circular-Green-economy Technology & Engineering Center (BCGeTEC), Department of Chemical Engineering, Faculty of Engineering, Chulalongkorn University, Bangkok 10330, Thailand

*Corresponding authors.

Email address: atthapon.sri@mahidol.edu (A. Srifa)

Entry ***Page No.***

Scheme SI. Schematic illustration of Ni–Pt, Ni, and Pt supported Al_2O_3 catalysts by a conventional wetness impregnation method………………………………………….... S3

Scheme SII. Schematic illustration of experimental set up for an evaluation of catalytic performance……………………………………………………………………….. S4

Table. S1 Operating conditions of a gas chromatography (GC) equipped using a flame ionization detector (FID)……………………………………………….………………... S5

Table. S2 Relative acidity distribution of reduced catalysts of NH_3-TPD………………... S6

Figure S1. N_2 adsorption-desorption isotherm and pore size distribution ……................. S7

Figure S2. Reusability experiment over $Ni_1Pt_{0.007}/Al_2O_3$ catalyst, X-ray diffraction patterns of reduced and spent $Ni_1Pt_{0.007}/Al_2O_3$ catalysts and thermogravimetric analysis profiles of reduced and spent $Ni_1Pt_{0.007}/Al_2O_3$ catalysts ………………………………………….…… S8

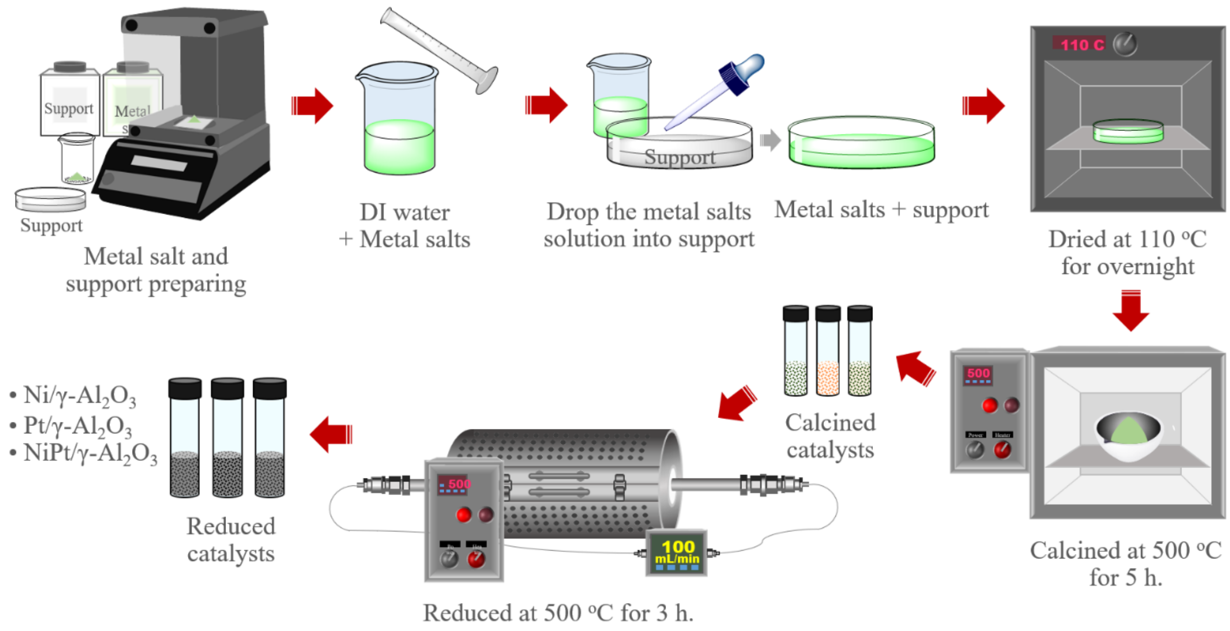

Scheme S1. Schematic illustration of Ni–Pt, Ni, and Pt supported Al_2O_3 catalysts by a conventional wetness impregnation method.

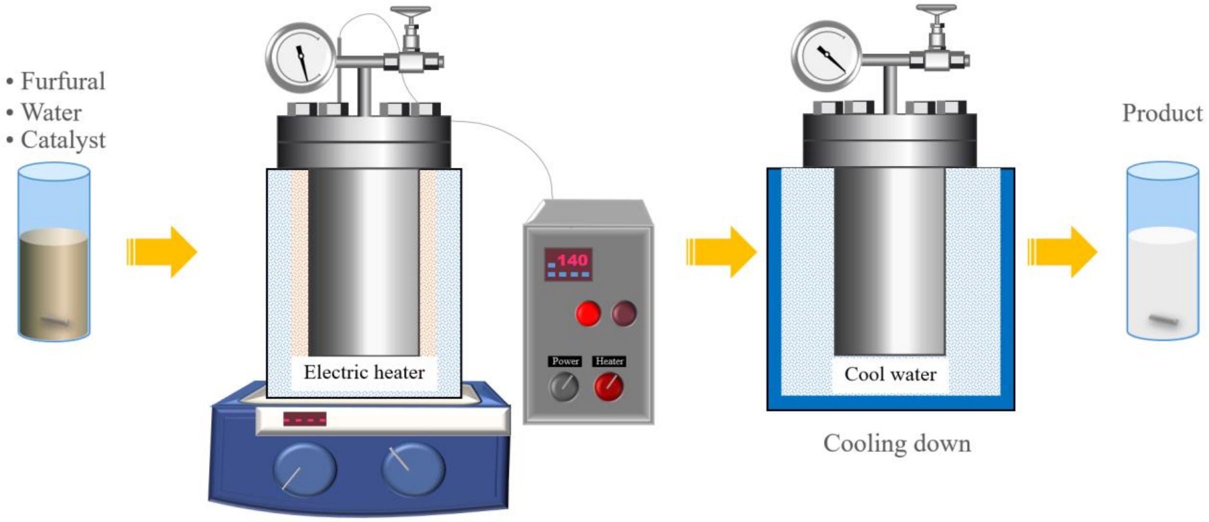

Scheme S2. Schematic illustration of experimental set up for an evaluation of catalytic performance.

Table. S1 Operating conditions of a gas chromatography (GC) equipped using a flame ionization detector (FID)

Model	GC 2014, Shimadzu
Integration	LabSolution.Ink software
Detector	FID
Column	Capillary CP-WAX-CB
Column length	30 m
Inside column diameter	0.25 mm
Film thickness	0.25 µm
Injector pressure program	30 kPa for 7 min, increased at 7 kPa min^{-1} to 120 kPa
Column temperature program	a) 50 ºC for 4 min, increased at 10 ºC min^{-1} to 160 ºC, held for 5 min<br>b) increased at 2 ºC min^{-1} to 170 ºC, held for 5 min<br>c) increased at 10 ºC min^{-1} to 200 ºC, held for 7 min
Detector temperature	230 ºC
Injector temperature	230 ºC
Carrier gas, inlet pressure	Helium, 30 kPa, total flow 17.9 mL min^{-1}, column flow 0.29 mL min^{-1}, initial linear velocity 9.3 cm s^{-1}, purge flow 3.0 mL min^{-1}
Split ratio	50
Injection volume	1 µL

Table. S2 Relative acidity distribution of reduced catalysts obtained by the results of NH_3-TPD.

Catalyst	Relative acidity distribution (%)			Total relative acidity distribution (%)
	Weak	Medium	Strong	
Ni/Al_2O_3	62.5	17.5	20	100
$Ni_1Pt_{0.007}/Al_2O_3$	71.3	11.25	6.25	88.8
Pt/Al_2O_3	40	6.25	3.75	50

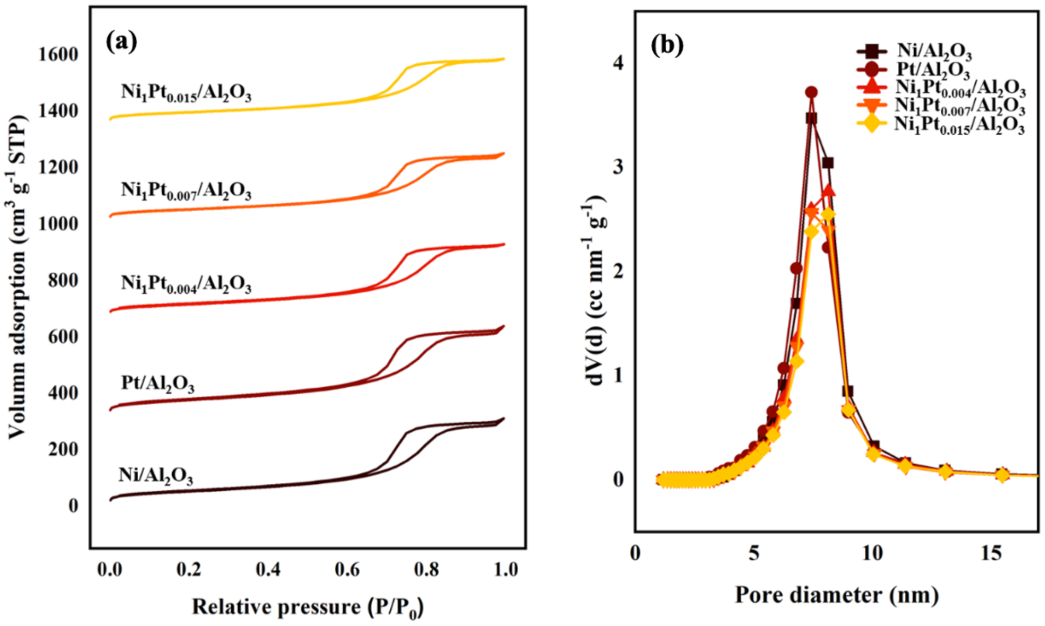

Figure S1. (a) N_2 adsorption-desorption isotherm and (b) pore size distribution of the calcined $Ni_1Pt_{0.015}/Al_2O_3$, $Ni_1Pt_{0.007}/Al_2O_3$, $Ni_1Pt_{0.004}/Al_2O_3$, Pt/Al_2O_3, and Ni/Al_2O_3 catalysts.

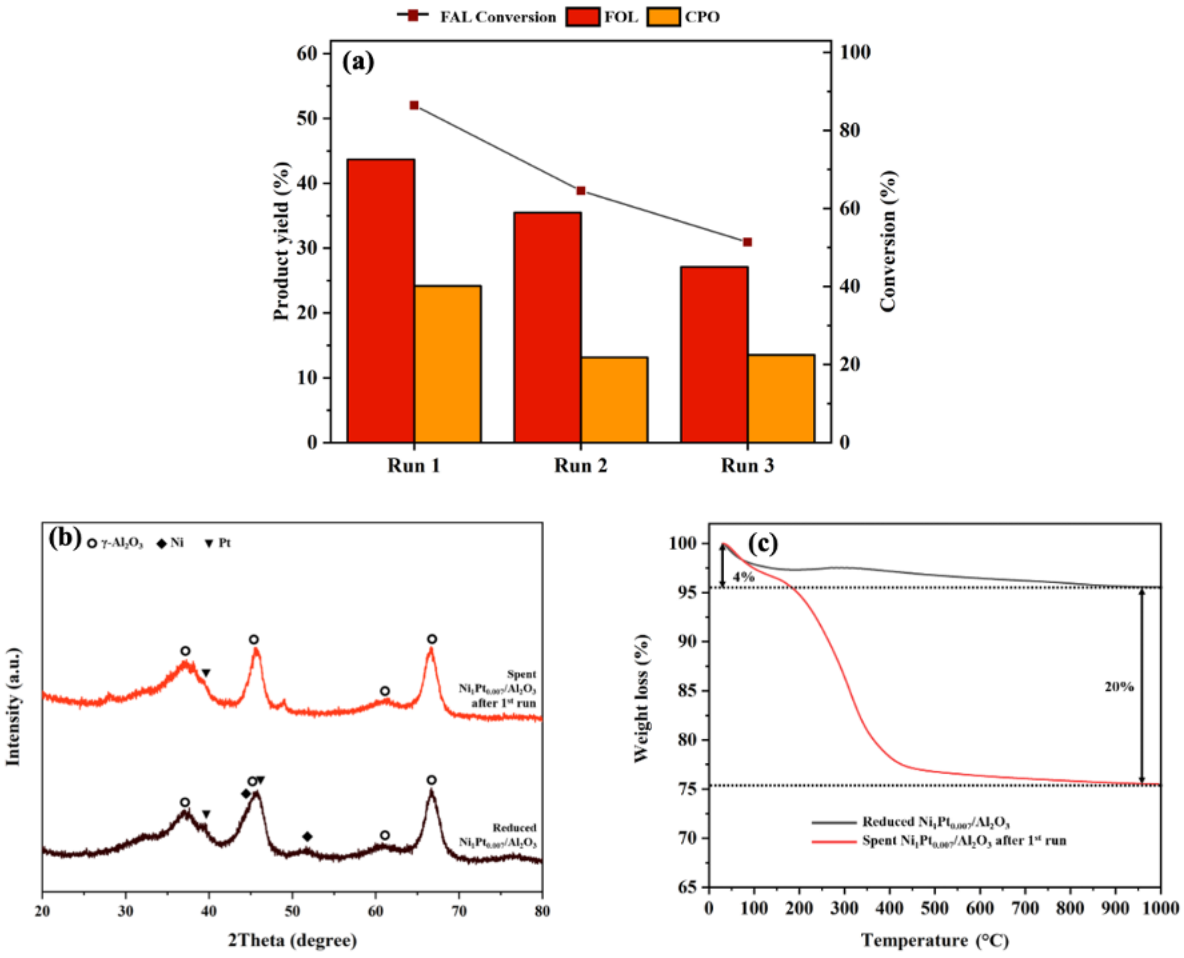

Figure S2. (a) Reusability experiment over $Ni_1Pt_{0.007}/Al_2O_3$ catalyst at a reaction temperature of 140 °C, 20 bars of H_2, and a reaction time for 2 h. The catalyst loading was 20 wt.% based on initial mass of furfural. All the experiments were conducted using 1 g of FAL feedstock in 40 g of water with 20% catalyst loading based on initial mass of FAL.; (b) X-ray diffraction patterns of reduced and spent $Ni_1Pt_{0.007}/Al_2O_3$ catalysts.; and (c) thermogravimetric analysis profiles of reduced and spent $Ni_1Pt_{0.007}/Al_2O_3$ catalysts under an air atmosphere.
